# Supplementary material for: The causal relationship between the human gut microbiota and pyogenic arthritis: a Mendelian randomization study
Source: Front Cell Infect Microbiol. 2024 Nov 26;14:1452480. doi: 10.3389/fcimb.2024.1452480 (PMC11629706; doi:10.3389/fcimb.2024.1452480)
Supplement: Supplementary file 1 [file DataSheet1.zip › mendelian test/ebi-a-GCST90027779.csv_forest.pdf]

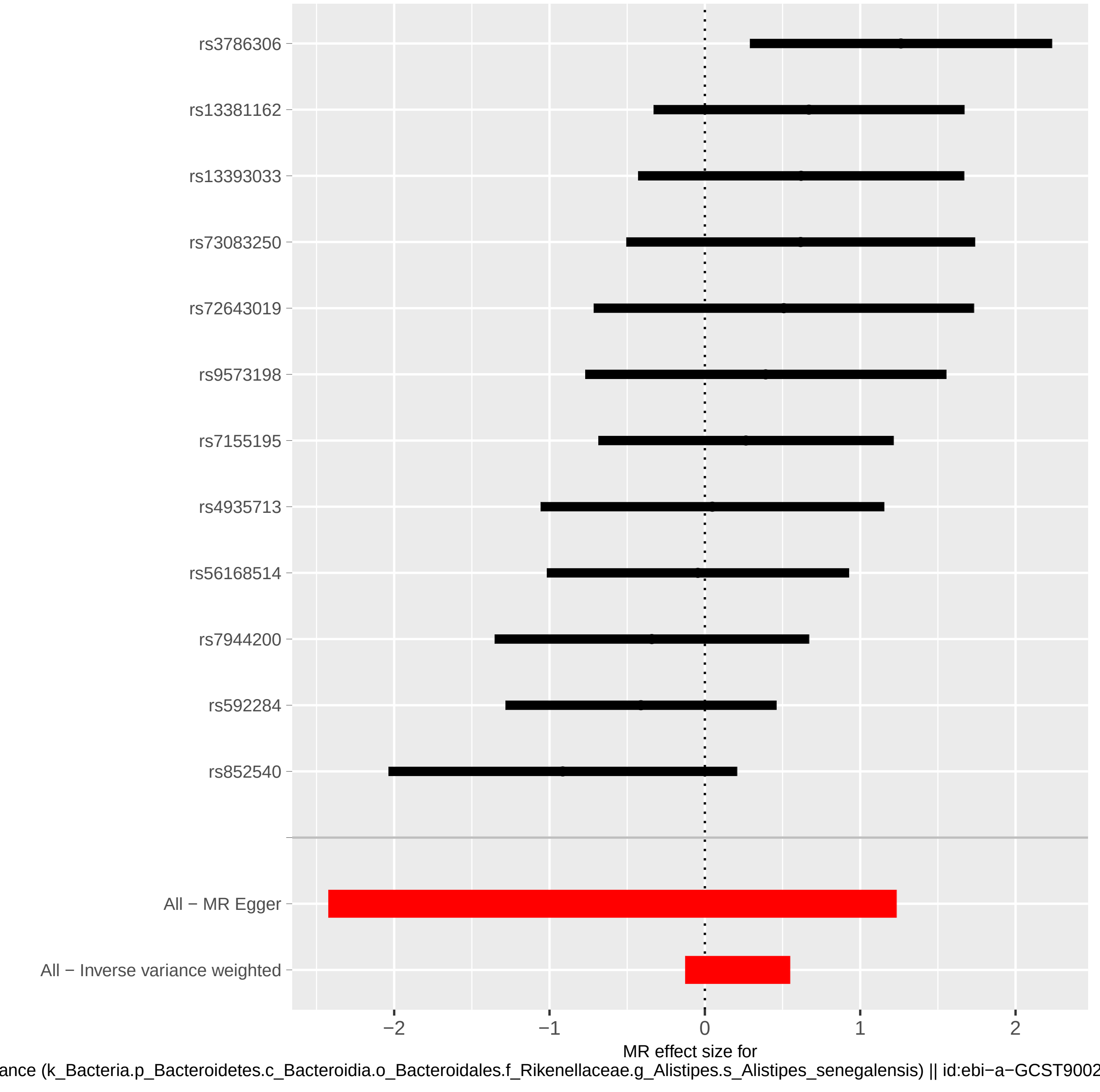
rs3786306
rs13381162
rs13393033
rs73083250
rs72643019
rs9573198
rs7155195
rs4935713
rs56168514
rs7944200
rs592284
rs852540
All − MR Egger
All − Inverse variance weighted
−2
−1
0
1
2
MR effect size for
ance (k_Bacteria.p_Bacteroidetes.c_Bacteroidia.o_Bacteroidales.f_Rikenellaceae.g_Alistipes.s_Alistipes_senegalensis) || id:ebi−a−GCST9002
